# Dosimetric predictors of hypothyroidism in oropharyngeal cancer patients treated with intensity-modulated radiation therapy

**DOI:** 10.1186/s13014-014-0269-4

**Published:** 2014-12-05

**Authors:** Arthur Chyan, Josephine Chen, Erin Shugard, Louise Lambert, Jeanne M Quivey, Sue S Yom

**Affiliations:** Department of Radiation Oncology, University of California, San Francisco, 1600 Divisadero Street, Suite H1031, San Francisco, CA 94115 USA

**Keywords:** Oropharyngeal cancer, Hypothyroidism, Intensity-modulated radiation therapy, Normal tissue toxicity

## Abstract

**Background:**

Radiation to the neck has long been associated with an elevated risk of hypothyroidism development. The goal of the present work is to define dosimetric predictors of hypothyroidism in oropharyngeal cancer (OPC) patients treated with intensity-modulated radiation therapy.

**Methods:**

Data for 123 patients, with a median follow up of 4.6 years, were retrospectively analyzed. Patients with elevated thyroid-stimulating hormone levels or with a clinical diagnosis were categorized as hypothyroid. Patient demographic parameters, thyroid volume, mean thyroid dose, the percent of thyroid volume receiving minimum specified dose levels (VxxGy), and the absolute thyroid volume spared from specified dose levels (VSxxGy) were analyzed. Normal-tissue complication probability (NTCP) was also calculated using several recently published models.

**Results:**

Thyroid volume and many radiation dosimetric parameters were statistically different in the hypothyroid group. For the patients with initial thyroid volumes of 8 cc or greater, several dosimetric parameters were found to define subgroups at statistically significant lower risk of developing hypothyroidism. Patients with VS45 Gy of at least 3 cc, VS50 Gy at least 5 cc, VS50 Gy at least 6 cc, V50 Gy below 45%, V50 Gy below 55%, or mean thyroid dose below 49 Gy had a 28-38% estimated risk of hypothyroidism at 3 years compared to a 55% risk for the entire study group. Patients with a NTCP of less than 0.75 or 0.8, calculated using recently published models, were also observed to have a lower risk of developing hypothyroidism.

**Conclusions:**

Based on long-term follow up data for OPC patients treated with IMRT, we recommend plan optimization objectives to reduce the volume of thyroid receiving over 45 Gy to significantly decrease the risk of developing hypothyroidism.

**Electronic supplementary material:**

The online version of this article (doi:10.1186/s13014-014-0269-4) contains supplementary material, which is available to authorized users.

## Background

The increased incidence of overt or subclinical hypothyroidism in patients who have received radiotherapy in the neck region has been documented for several decades [1-5]. While there are patient-intrinsic risk factors, such as sex and race, that have been shown to influence hypothyroidism risk [6], radiation dosimetric treatment parameters are the only potential risk factors that can be controlled by the radiation oncology team by modifying treatment techniques. There is now a large body of research investigating the relationship between radiation dose and hypothyroidism development in various patient populations [1,4,7-16]. The results have been heterogeneous and there remains a lack of consensus on the dose–response curve. The present work focuses on the incidence of hypothyroidism in oropharyngeal cancer patients treated with modern intensity-modulated radiation therapy (IMRT) techniques, and provides the longest median follow up reported to date. The goal of the study was to define the dosimetric parameters predictive of an increased risk of developing hypothyroidism for this patient cohort in order to guide future planning objectives.

## Methods

### Patients and hypothyroidism determination

Between June 2000 and February 2011, 286 patients with squamous cell carcinoma of the oropharynx were treated at the University of California – San Francisco (UCSF) with definitive or adjuvant radiation therapy with or without chemotherapy and/or surgery. UCSF radiation oncology medical records for these patients were retrospectively reviewed. This study was approved by the UCSF institutional review board.

After review of the available records, 123 patients were included in the study. Patients were not included due to (1) previous thyroid disorders or thyroid surgery [11 patients], (2) treatment break extending beyond seven days or inability to complete treatment [4 patients], (3) recurrence within 1 year of treatment or death within two years of radiation treatment’s end date [25 patients], (4) insufficient treatment plan data [26 patients], and (5) insufficient follow-up data to determine post-treatment thyroid status [97 patients]. For patients with insufficient follow up records, phone contact was attempted to obtain current thyroid status updates.

Patients were categorized as having remained euthyroid or as having developed overt hypothyroidism or subclinical hypothyroidism based on clinical and laboratory values for thyroid-stimulating hormone (TSH) and thyroxine (T4). Standard clinical follow up after completion of radiation was every 3 months for the first 2 years, every 6 months up to year 5 and then yearly. Thyroid hormone testing was done every 6 months for the first 2 years and then yearly unless the patient presented with clinical symptoms. However, clinical follow up and hormone testing was not uniform for all patients. The euthyroid classification was chosen if the patient’s record indicated that TSH (0.4-4.0 mIU/L) and T4 (9–24 pmol/L) levels were within normal limits, there was no manifestation of clinical symptoms associated with altered thyroid status, and there was no thyroid replacement medication use/prescription. Overt hypothyroidism was designated if the attending physician diagnosed hypothyroidism and supplemental thyroid hormone was used/prescribed. Subclinical cases were situations where thyroid medication status could not be confirmed but laboratory test results showed elevated TSH and reduced T4 levels; these were relatively few in number (9 patients or 7%). All cases of hypothyroidism were presumed to be primary hypothyroidism as doses to the pituitary were low in this population of exclusive OPC patients (mean pituitary dose of 3.9 Gy, range 0.1 - 16.1 Gy).

### Radiation treatment

All 123 patients were treated with daily IMRT over 30–36 fractions. Most patients were treated at 2.12 Gy per fraction for 33 fractions to a total of 69.96 Gy to the GTV. Elective nodal regions in the neck were generally treated to 54.1-59.4 Gy. Because planning objectives to reduce the thyroid dose were not used for most patients, many patients received large radiation doses to the thyroid, with significant portions of the gland receiving 50 Gy or more. Radiation treatments were planned on the Corvus treatment planning system (Best Nomos, Pittsburgh, Pennsylvania, United States) or the Pinnacle treatment planning system (Philips Healthcare, Amsterdam, The Netherlands). Radiation treatment was delivered in the form of 6 MV photon beams via linear accelerator. Gantry angle number ranged from five to nine.

### Contouring and thyroid data collection

Each treatment plan was transferred to the MIM contouring system (MIM Software, Cleveland, Ohio, United States), and the thyroid gland was manually contoured by the same individual for all plans. Thyroid data were extracted from MIM, which included thyroid gland volume, mean dose to the thyroid, *percent* volume of thyroid gland receiving various minimum dose levels (designated as VxxGy(%)), and the *absolute* volume of thyroid gland *spared* from various dose levels (designated as VSxxGy(cc)). For example, the absolute volume spared 40 Gy is equal to the total thyroid volume subtracted by the absolute volume that received at least 40 Gy (VSxxGy(cc) = ThyroidVol(cc) – VxxGy(cc)).

### Statistical analysis

The subclinically hypothyroid patients were grouped with the overtly hypothyroid patients to enable comparison with the euthyroid group. Univariate analysis was conducted to assess variables associated with developing post-radiation hypothyroidism. Multivariate logistic regression models were applied to test the associations when multiple factors were considered. Kaplan-Meier curves describing freedom from hypothyroidism as a function of post-irradiation time and the log-rank test were also used to analyze and compare the risk of developing hypothyroidism for the entire study population and various subgroups. Subgroups containing less than 10% of the total study population were not analyzed. Subgroups defined by normal tissue complication probability (NTCP) models proposed in the literature were also analyzed.

## Results

### Patient outcomes

Table 1 summarizes the patient characteristics of the study population. The median duration of follow up was 4.6 years. At time of the analysis, 75 of the 123 patients (61%) had developed hypothyroidism or subclinical hypothyroidism. For those who developed hypothyroidism, the median time to development was 1.7 years. The overall survival rate for the study group was 98% at 2 and 4 years post-radiation. These high survival rates, however, are in part due to the selection criteria for the study. Patients who recurred or died soon after treatment were excluded since hypothyroidism development typically has a 1–2 year latency period.

**Table 1:** **Patient and treatment characteristics**

**Characteristic**	**Number**
N	123
Age (y), median (range)	59 (35–86)
Sex	
Female	18 (15%)
Male	105 (85%)
Race	
Asian/Pacific Islander	6 (5%)
Black	10 (8%)
Latino/Hispanic	3 (2%)
White	98 (80%)
Other	6 (5%)
Stage	
II	4 (3%)
III	21 (17%)
IVA	93 (76%)
IVB	5 (4%)
Oropharynx site	
Tonsil	48 (39%)
Base of tongue	69 (56%)
Soft palate	3 (2%)
Pharyngeal wall	1 (1%)
Not specified	2 (2%)
Treatment modality	
IMRT^1^ alone	12 (10%)
IMRT + chemotherapy^2^	84 (68%)
IMRT + surgery^3^	9 (7%)
IMRT + chemotherapy^2^ + surgery^3^	18 (15%)
Tumor volume (cm^3^), mean	104.51
Standard deviation	80.84
Pre-radiation thyroid volume (cc), mean (SD)	13.22 (10.44)
Post-radiation thyroid status	
Euthyroid	48 (39%)
Overt Hypothyroidism	66 (54%)
Subclinical Hypothyroidism	9 (7%)

^1^IMRT = intensity-modulated radiation therapy.

^2^chemotherapy = cisplatin, carboplatin with paclitaxel, and/or cetuximab; induction regimens given in a small number of patients also included docetaxel and/or fluorouracil.

^3^surgery = pre-radiation tonsillectomy, parotidectomy, glossopharyngectomy, epiglottis resection, and/or base of tongue resection.

### Thyroid volume analysis and stratification

**Figure 1:**
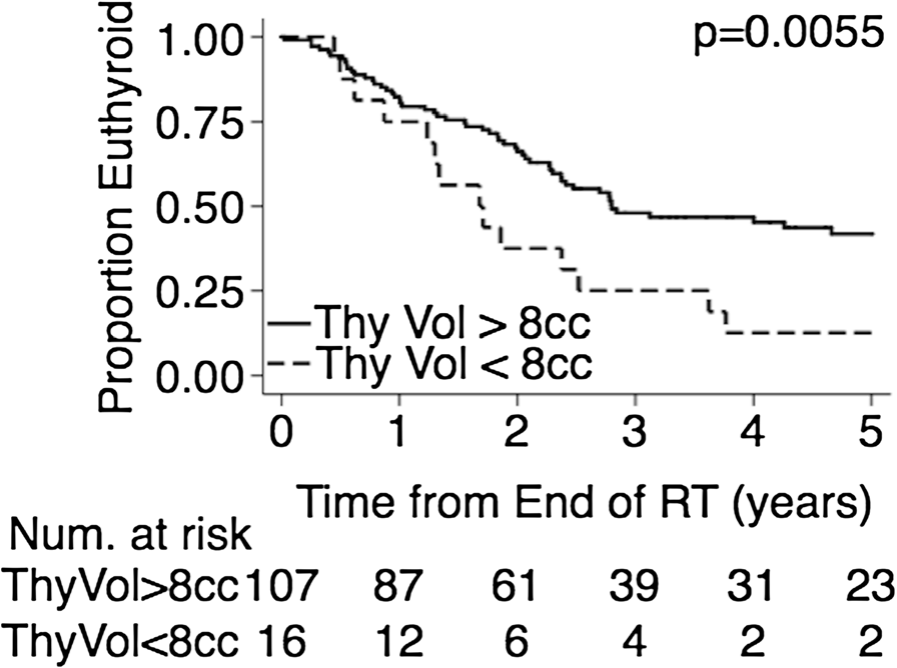
**Comparison of Kaplan-Meier curves estimating freedom from hypothyroidism for patients with initial thyroid volume ≥8 cc and for patients with thyroid volume <8 cc in years following completion of radiation therapy.**

An initial univariate analysis comparing the euthyroid and hypothyroid groups was conducted (Additional file 1: Table S1). Multivariate logistic models were also explored. Additional file 2: Table S2 provides an example. Although the pre-treatment thyroid volume did not demonstrate statistical significance in the multivariate models, it was significant in the univariate analysis and many investigators have noted an association between thyroid volume and hypothyroidism [8,9,14,16]. This prompted us to stratify the entire study group by pre-irradiation thyroid size. Using the Kruskal-Wallis test and the log-rank test, subgroups based on pre-irradation thyroid size were compared. The volume cut-off used to separate the patients was varied in 1 cc increments. An 8 cc thyroid volume threshold was found to yield the lowest p-values (0.004 for Kruskal-Wallis and 0.0055 for log-rank), and thus defined two groups with highly significant differences in hypothyroidism risk in this study. An estimated 45% of the patients in the entire study population (95% confidence interval of 35.3-54.0%) remained free from hypothyroidism after a follow-up period of three years. As seen in Figure 1, 48.5% (95% CI of 38.0-58.0%) of the patients with pre-irradiation thyroid volumes ≥ 8 cc remained free from hypothyroidism after 3 years, while only 25.0% (95% CI of 8.0-47.5%) of patients with thyroid volumes < 8 cc had normal thyroid function after 3 years. The high dose levels and non-utilization of thyroid planning constraints for patients in this study put those with small thyroid volumes at considerable risk of developing hypothyroidism. It would be difficult to define a threshold or critical dose for this group as it was likely exceeded for all patients. Therefore, for subsequent analysis, the 16 patients with initial thyroid volumes < 8 cc were excluded to better focus on what relationship could be found between dosimetric parameters and hypothyroidism development for patients with moderate to larger initial thyroid volumes (107 patients).

### Hypothyroidism risk for patients with initial thyroid volumes of at least 8 cc

Table 2 summarizes the univariate analysis comparing the euthyroid and hypothyroid groups for patients with an initial thyroid volume of at least 8 cc. As seen in Table 2, none of the general patient demographic parameters were significantly different between the two groups. Unlike the results of the univariate analysis on the entire study group (Additional file 1: Table S1), thyroid volume was not significantly different between the euthyroid and hypothyroid groups once the patients with very small thyroid volumes were removed. Numerous dosimetric parameters were significantly different between the euthyroid and hypothyroid groups. Many of the various dosimetric parameters were highly correlated to each other (p values below 0.01).

**Table 2:** **Univariate analysis for patients with thyroid volume of at least 8 cc**

**Variable**	**Euthyroid**	**Hypothyroid**	**p-Value**
	**(n = 47)**	**(n = 60)**	
Gender			0.22^2^
F	3	9	
M	44	51	
Age (y), mean; [SD]	60.3 [8.2]	57.8 [10.8]	0.16^1^
Weight (lbs), mean; [SD]	183 [35.7]	178 [38.4]	0.46^1^
T Stage			0.20^1^
1	12	14	
2	24	22	
3	8	18	
4	3	6	
N Stage			0.77^1^
0	3	7	
1	10	7	
2	30	45	
3	4	1	
Neck Dissection			0.29^2^
No	42	48	
Yes	5	12	
Chemo			1.00^2^
No	8	10	
Yes	39	50	
Follow-up time (m), mean; [SD]	52.1 [30.3]	57.0 [24.9]	0.23^1^
Thyroid volume (cc), mean; [SD]	15.6 [15.5]	13.1 [4.6]	0.49^1^
Mean dose (Gy), mean; [SD]	52.8 [11.7]	57.8 [7.3]	0.048^1^
V10 (%), mean; [SD]	97.4 [12.1]	99.7 [2.5]	0.048^1^
V20 (%), mean; [SD]	94.4 [16.3]	99.2 [4.3]	0.038^1^
V30 (%), mean; [SD]	91.7 [20.2]	98.2 [7.2]	0.015^1^
V40 (%), mean; [SD]	85.6 [24.4]	94.9 [13.5]	0.067^1^
V50 (%), mean; [SD]	70.9 [31.5]	84.2 [22.2]	0.065^1^
V60 (%), mean; [SD]	34.8 [31.3]	43.7 [28.8]	0.075^1^
V70 (%), mean; [SD]	2.86 [4.95]	7.1 [12.4]	0.026^1^
VS10 (cc), mean; [SD]	0.36 [1.53]	0.05 [0.39]	0.048^1^
VS20 (cc), mean; [SD]	0.75 [2.01]	0.108 [0.638]	0.037^1^
VS30 (cc), mean; [SD]	1.09 [2.51]	0.225 [0.976]	0.016^1^
VS40 (cc), mean; [SD]	1.89 [3.12]	0.683 [1.8]	0.057^1^
VS50 (cc), mean; [SD]	4.08 [4.72]	2.07 [3.03]	0.048^1^
VS60 (cc), mean; [SD]	9.83 [9.1]	7.38 [4.8]	0.10^1^
VS70 (cc), mean; [SD]	15.2 [15.0]	12.2 [4.65]	0.19^1^

^1^based on Mann–Whitney test.

^2^based on Fisher’s exact test.

A multivariate analysis was performed to test the association of the dosimetric parameters with hypothyroidism development if thyroid volume and other patient demographic parameters were considered simultaneously. Because the dosimetric parameters were highly correlated with each other, the multivariate models only included one dosimetric parameter at a time. The dosimetric parameter, ideal body weight, thyroid volume, age at time of treatment, and sex were entered into the multivariate logistic regression model. For this specific analysis, data from 84 patients that had recorded ideal body weight information were used. None of the non-dosimetric parameters were found to have significant p values (≤ 0.05) in any of the multivariate models. The dosimetric parameters with significant p values were V30 Gy (p value 0.038), V40 Gy (p value 0.017), V50 Gy (p value 0.025), mean dose (p value 0.017), VS30 Gy (p value 0.046), VS40 Gy (p value 0.026), and VS50 Gy (p value 0.036). Table 3 displays the regression parameters for the V40 Gy model. Similar results were found for the models utilizing the other significant dosimetric parameters. Because the multivariate analysis did not show any benefit from the addition of the demographic parameters, subsequent analysis focused on the dosimetric variables.

**Table 3:** **V40 Gy multivariate model for patients with thyroid volume of at least 8 cc**

**Variable**	**Odds ratio**	**Lower 95% CI**	**Upper 95% CI**	**p-Value**
V40Gy	1.03	1.01	1.06	0.017
Ideal Body Weight	0.95	0.89	1.03	0.21
Thyroid Volume	0.95	0.86	1.04	0.24
Age at Treatment	0.98	0.93	1.03	0.39
Sex (M)	0.45	0.05	4.4	0.49

**Figure 2:**
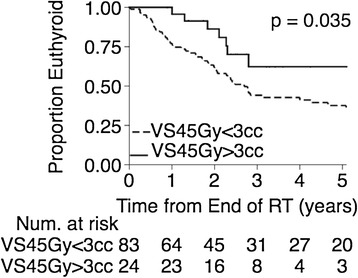
**Kaplan-Meier curves of freedom from hypothyroidism for subgroups of the patients with pre-radiation thyroid volumes ≥8 cc.** The lower incidence group had thyroid volumes spared 45 Gy of ≥3 cc and the higher incidence group had volumes spared 45 Gy <3 cc.

For the 107 patients with initial thyroid volumes of at least 8 cc, comparisons were made using various dosimetric threshold values using the log-rank test. The dosimetric variables and the threshold values that showed significant differences in hypothyroidism development between the two groups are shown in Table 4. The Kaplan-Meier curve for VS45 Gy with a threshold value of 3 cc is shown in Figure 2. The other dosimetric parameters and associated thresholds resulted in very similar groupings of patients with similar Kaplan-Meier curves. The patients in the lower dose groups as defined by the parameters in Table 4 had a three-year freedom from hypothyroidism rate that ranged from 62% to 72%, as opposed to 45% observed for the entire study population.

**Table 4:** **Dosimetric criteria associated with lower risk for hypothyroidism in patients with pre-irradiation thyroid volumes ≥ 8 cc**

**Dosimetric parameter**	**Low-risk group definition**	**Log-rank p value**	**3-year freedom from HT**	**Lower 95% CI**	**Upper 95% CI**
VS45 Gy	≥3 cc	0.035	65.0%	36.0%	82.5%
VS50 Gy	≥5 cc	0.037	65.0%	36.0%	82.5%
VS50 Gy	≥6 cc	0.035	61.5%	35.5%	80.5%
V50 Gy	<45%	0.041	71.5%	39.2%	88.5%
V50 Gy	<55%	0.050	69.5%	41.0%	86.3%
Mean Dose	<49 Gy	0.041	71.0%	39.5%	88.5%

**Figure 3:**
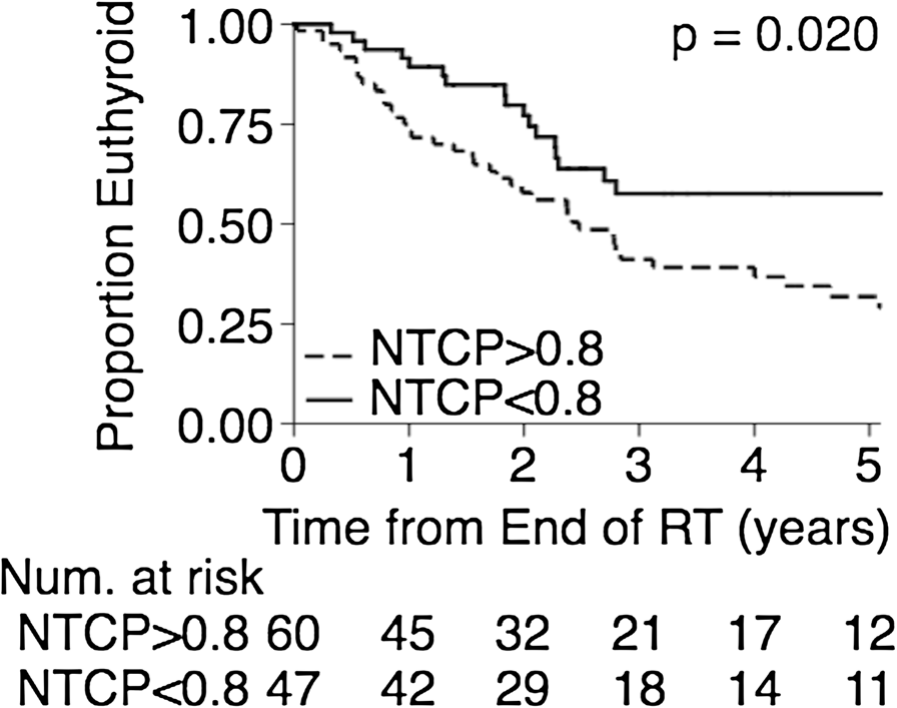
**Kaplan-Meier curves of freedom from hypothyroidism for subgroups of the patients with pre-radiation thyroid volumes ≥8 cc.** The lower incidence group had NTCP < 0.8 and the higher incidence group had NTCP > 0.8, based on the mixture model reported in Ronjom et al. [16].

Recently, several normal tissue complication probability (NTCP) models have been proposed for radiation-induced hypothyroidism development [13,14,16]. NTCP was calculated for the patients in this study using proposed formulas. As was performed for the dosimetric parameters, for the patients with initial thyroid volume of at least 8 cc (107 patients), risk of hypothyroidism for subgroups defined by threshold values of NTCP were compared using the log-rank test. The NTCP threshold value was varied in 0.05 increments, and the threshold value that yielded the lowest p-value in the log-rank test was found. The NTCP calculated using the mixture model formula proposed by Ronjom et al. [16] with a threshold of 0.8 defined subgroups with significant differences in hypothyroidism development (p value 0.02). Patients with NTCP < 0.8 had a three-year freedom from hypothyroidism rate of 58.0% (95% CI of 41.0-72.0%), as displayed in Figure 3. The NTCP calculated using the formula proposed by Boomsma et al. [14] with a threshold of 0.75 also separated the patients into groups with different risks of hypothyroidism development (p value 0.02). Patients with NTCP < 0.75 had a three-year freedom from hypothyroidism rate of 55.0% (95% CI of 39.5-68.5%). The Kaplan-Meier curves are similar to those displayed in Figure 3.

## Discussion

### Patient outcomes

In this retrospective study of oropharyngeal cancer patients treated with IMRT, 61% of patients developed overt or subclinical hypothyroidism after a median follow up of 4.6 years. This is somewhat higher than the 47.7% incidence reported by Diaz et al. [9] for a group of head-and-neck patients also treated with IMRT. However, median follow up for that study was lower, at only 1.08 years. Other studies of hypothyroidism in head-and-neck patients treated with either a mix of IMRT and three-dimensional (3D) techniques or 3D exclusively, have reported wide ranges of hypothyroidism incidence. In this study, all patients were treated with IMRT, but patients were planned without using objectives to reduce the dose to the thyroid. As noted by Diaz et al., the use of IMRT without a specific constraint of the thyroid during planning leads to higher thyroid doses and to a high incidence of hypothyroidism. This was also evident in our study population.

### Effect of non-dosimetric factors

In this study, the only non-dosimetric parameter to be found to be significantly different between the euthyroid and hypothyroid groups was pre-treatment thyroid volume. Other studies have found other parameters, including female sex, to be associated with higher risk of hypothyroidism [6]. The low number of women in our patient population (18/123) likely reduced the effect of sex to the insignificant level in our analysis.

Similar to many other studies [8,9,14,16], the initial thyroid volume was found to be significantly associated with risk of developing post-radiation hypothyroidism in our study. This was one of the motivating factors for analyzing the absolute spared thyroid volume, defined as the volume receiving less than a particular dose. The significance of absolute thyroid volume suggested the possibility that an absolute volume of functional thyroid might be necessary to avoid hypothyroidism. Absolute spared thyroid volumes had previously been found to be predictive of hypothyroidism development in Hodgkin’s lymphoma patients [13], but had not been analyzed for head-and-neck cancer patients. However, because there was such a strong association of hypothyroidism with the initial thyroid volume and the doses received by our patients were so high, almost all of the patients with very small thyroids developed hypothyroidism. Thus, it was not feasible to find the correct threshold dosimetric values for these patients. Inclusion of these patients in the dosimetric analysis could have potentially produced misleading results by introducing a systematic bias for the dosimetric parameters computed using absolute volumes. It was decided to exclude patients below the threshold initial thyroid volume of 8 cc in subsequent dosimetric analyses and focus on patients with moderate and larger thyroid volumes. The choice of 8 cc as a volume threshold was determined by looking at hypothyroidism development in our study population. However, this is likely not an exact biological threshold volume as our results may be affected by the peculiarities of our study. To effectively study the dose and volume effect for patients with smaller thyroid volumes would require larger numbers of such patients who received differing dose levels and had a variety of responses.

### Dosimetric predictors of hypothyroidism

Although the study by Diaz et al. of head-and-neck cancer patients treated with IMRT did not find dosimetric parameters significantly associated with hypothyroidism risk [9], in our current, study several dosimetric parameters showed a statistically significant relationship in the univariate and multivariate analysis and in the subsequent Kaplan-Meier analysis of patients with initial thyroid volumes of at least 8 cc. All the significant variables, listed in Table 4, suggest a benefit to reducing the volume of thyroid that receives approximately 45 Gy. Interestingly, the meta-analysis by Vogelius estimated a 50% risk of hypothyroidism for a mean dose of 45 Gy [6], and a NTCP model predicting elevated TSH in head-and-neck cancer patients at 1 year post-radiation also found a D50 of 44 Gy [15]. However, other studies have suggested lower dose levels are important. Studies of Hodgkin’s lymphoma patients have found an increased risk when the thyroid received doses over 30 Gy [1,12], although these observations may be complicated by the use of lymphangiogram dye in lymphoma patients [17]. These low doses were not delivered in our cohort, and thus it is possible that additional reductions in the incidence of hypothyroidism would be obtained by reducing volumes receiving doses above 30 Gy in our patients. However, in terms of dosimetric objectives that are reasonably achievable for oropharyngeal cancer patients treated with IMRT, we have observed a significant reduction in hypothyroidism rates at 3 years post-radiation if at least 3 cc of thyroid can be spared from doses exceeding 45 Gy, in patients with initial thyroid volumes of at least 8 cc. Additional benefit from further reduction in thyroid doses is likely, but could not be confirmed with the current data set. Thus this research provides a realistic set of planning goals and expected outcomes for clinicians in counseling and treating oropharyngeal cancer patients.

The NTCP values calculated using the mixture model formula from Ronjom et al. [16] and the model proposed by Boomsma et al. [14] were also found to define subgroups at higher and lower risk of hypothyroidism development in this study. Both models were based on data from head-and-neck patients. The calculated NTCP for most of the patients in this study was high, the median NTCP was 0.89 and 0.79 using the Ronjom formula and Boomsma formula respectively. This is not surprising given the high doses delivered to the thyroid, and qualitatively corresponds with the observed outcome that a large percentage of patients developed hypothyroidism. In this study, the ability of the NTCP models to stratify patients for risk of hypothyroidism development seemed comparable to the use of simple dosimetric parameters and thresholds. However, there may be more advantage to NTCP models for patient groups with more heterogeneity in the delivered doses and thus a more even spread of patients with low and high NTCP values. As thyroid dose constraints are incorporated into routine clinical practice, delivered doses will decrease and there will be an opportunity to study the incidence of hypothyroidism in oropharyngeal patients who receive lower thyroid doses. Thus future studies should have an enhanced ability to investigate the dose-volume response relationship for hypothyroidism and provide validation for robust NTCP models.

## Conclusions

This study provides a comprehensive analysis of dosimetric predictors for hypothyroidism development in oropharyngeal cancer patients treated with definitive IMRT, utilizing long-term follow up data from 123 patients. A significant reduction of hypothyroidism rates at 3 years post-RT (38% incidence versus 55%) was found for patients with initial thyroid volumes over 8 cc and at least 3 cc of thyroid spared radiation doses of 45 Gy or more. Similar patient stratification was obtained using related dosimetric parameters and thresholds, such as mean thyroid dose less than 49 Gy. To reduce the risk of hypothyroidism in these patients, it is recommended that IMRT optimization objectives be used to reduce the volume of thyroid receiving 45 Gy. Stricter criteria may be necessary for patients with smaller (< 8 cc) initial thyroid volumes.

## Additional files

Additional file 1: Table S1. Univariate analysis for all patients. Additional file 2: Table S2. V40 Gy Multivariate Model for all Patients.

## References

[1] Hancock SL, Cox RS, McDougall IR (1991). Thyroid diseases after treatment of Hodgkin’s disease. N Engl J Med.

[2] Weissler MC, Berry BW (1991). Thyroid-stimulating hormone levels after radiotherapy and combined therapy for head and neck cancer. Head Neck.

[3] Grande C (1992). Hypothyroidism following radiotherapy for head and neck cancer: multivariate analysis of risk factors. Radiother Oncol.

[4] Kuten A, Lubochitski R, Fishman G, Dale J, Stein ME (1996). Postradiotherapy hypothyroidism: radiation dose response and chemotherapeutic radiosensitization at less than 40 Gy. J Surg Oncol.

[5] Tell R, Sjodin H, Lundell G, Lewin F, Lewensohn R (1997). Hypothyroidism after external radiotherapy for head and neck cancer. Int J Radiat Oncol Biol Phys.

[6] Vogelius IR, Bentzen SM, Maraldo MV, Petersen PM, Specht L (2011). Risk factors for radiation-induced hypothyroidism: a literature-based meta-analysis. Cancer.

[7] Bhandare N, Kennedy L, Malyapa RS, Morris CG, Mendenhall WM (2007). Primary and central hypothyroidism after radiotherapy for head-and-neck tumors. Int J Radiat Oncol Biol Phys.

[8] Alterio D, Jereczek-Fossa BA, Franchi B, D'Onofrio A, Piazzi V, Rondi E, Ciocca M, Gibelli B, Grosso E, Tradati N, Mariani L, Boboc GI, Orecchia R (2007). Thyroid disorders in patients treated with radiotherapy for head-and-neck cancer: a retrospective analysis of seventy-three patients. Int J Radiat Oncol Biol Phys.

[9] Diaz R, Jaboin JJ, Morales-Paliza M, Koehler E, Phillips JG, Stinson S, Gilbert J, Chung CH, Murphy BA, Yarbrough WG, Murphy PB, Shyr Y, Cmelak AJ (2010). Hypothyroidism as a consequence of intensity-modulated radiotherapy with concurrent taxane-based chemotherapy for locally advanced head-and-neck cancer. Int J Radiat Oncol Biol Phys.

[10] Lin Z, Wu VW, Lin J, Feng H, Chen L (2011). A longitudinal study on the radiation-induced thyroid gland changes after external beam radiotherapy of nasopharyngeal carcinoma. Thyroid.

[11] Johansen S, Reinertsen KV, Knutstad K, Olsen DR, Fossa SD: **Dose distribution in the thyroid gland following radiation therapy of breast cancer – a retrospective study.** *Radiat Oncol* 2011, **6:**68. 10.1186/1748-717X-6-68 PMC3128838 21651829

[12] Cella L, Conson M, Caterino M, De Rosa N, Liuzzi R, Picardi M, Grimaldi F, Solla R, Farella A, Salvatore M, Pacelli R (2012). Thyroid V30 predicts radiation-induced hypothyroidism in patients treated with sequential chemo-radiotherapy for Hodgkin’s lymphoma. Int J Radiat Oncol Biol Phys.

[13] Cella L, Liuzzi R, Conson M, D'Avino V, Salvatore M, Pacelli R: **Development of multivariate NTCP models for radiation-induced hypothyroidism: a comparative analysis.** *Radiat Oncol* 2012, **7:**224. 10.1186/1748-717X-7-224 PMC3573930 23270411

[14] Boomsma MJ, Bijl HP, Christianen ME, Beetz I, Chouvalova O, Steenbakkers RJ, van der Laan BF, Wolffenbuttel BH, Oosting SF, Schilstra C, Langendijk JA (2012). A prospective cohort study on radiation-induced hypothyroidism: development of an NTCP model. Int J Radiat Oncol Biol Phys.

[15] Bakhshandeh M, Hashemi B, Mahdavi SR, Nikoofar A, Vasheghani M, Kazemnejad A (2013). Normal tissue complication probability modeling of radiation-induced hypothyroidism after head-and-neck radiation therapy. Int J Radiat Oncol Biol Phys.

[16] Ronjom MF, Brink C, Bentzen SM, Hegedus L, Overgaard J, Johansen J (2013). Hypothyroidism after primary radiotherapy for head and neck squamous cell carcinoma: Normal tissue complication probability modeling with latent time correction. Radiother Oncol.

[17] Fein DA, Hanlon AL, Corn BW, Curran WJ, Coia LR (1996). The influence of lymphangiography on the development of hypothyroidism in patients irradiated for Hodgkin’s disease. Int J Radiat Oncol Biol Phys.

