# Supplementary material for: Dosimetric predictors of hypothyroidism in oropharyngeal cancer patients treated with intensity-modulated radiation therapy
Source: Radiat Oncol. 2014 Dec 5;9:269. doi: 10.1186/s13014-014-0269-4 (PMC4265326; doi:10.1186/s13014-014-0269-4)
Supplement: Additional file 1: Table S1. — Univariate analysis for all patients. [file 13014_2014_269_MOESM1_ESM.doc]

Additional file 1: Table S1. Univariate analysis for all patients

| **Variable** | **Euthyroid**  (n = 48) | **Hypothyroid**  (n = 75) | **p-Value** |
| --- | --- | --- | --- |
| Gender  F  M | 4  44 | 14  61 | 0.13^2^ |
| Age (y), mean; [SD] | 60.3 [8.1] | 59.1 [10.8] | 0.44^1^ |
| Weight (lbs), mean; [SD] | 182 [35.9] | 175 [37.9] | 0.28^1^ |
| T Stage  1  2  3  4 | 12  24  8  4 | 18  28  21  8 | 0.30^1^ |
| N Stage  0  1  2  3 | 4  10  30  4 | 9  9  56  1 | 0.84^1^ |
| Neck Dissection  No  Yes | 43  5 | 60  15 | 0.21^2^ |
| Chemo  No  Yes | 8  40 | 12  63 | 1.00^2^ |
| Follow-up time (m), mean; [SD] | 53 [30] | 58 [25] | 0.21^1^ |
| Thyroid volume (cc), mean; [SD] | 15.4 [15.4] | 11.8 [4.85] | 0.024^1^ |
| Mean dose (Gy), mean; [SD] | 53 [11.7] | 57.4 [8.4] | 0.080^1^ |
| V10 (%), mean; [SD] | 97.4 [12.0] | 99 [6.6] | 0.077^1^ |
| V20 (%), mean; [SD] | 94.5 [16.1] | 98.4 [9.12] | 0.039^1^ |
| V30 (%), mean; [SD] | 91.8 [20.0] | 97.5 [11.7] | 0.007^1^ |
| V40 (%), mean; [SD] | 85.9 [24.2] | 94.5 [16.1] | 0.053^1^ |
| V50 (%), mean; [SD] | 71.4 [31.4] | 84.6 [22.6] | 0.060^1^ |
| V60 (%), mean; [SD] | 35.7 [31.6] | 42.8 [29.0] | 0.140^1^ |
| V70 (%), mean; [SD] | 2.85 [4.89] | 6.21 [11.8] | 0.140^1^ |
| VS10 (cc), mean; [SD] | 0.355 [1.52] | 0.0887 [0.541] | 0.075^1^ |
| VS20 (cc), mean; [SD] | 0.735 [1.99] | 0.151 [0.791] | 0.037^1^ |
| VS30 (cc), mean; [SD] | 1.07 [2.49] | 0.256 [1.08] | 0.007^1^ |
| VS40 (cc), mean; [SD] | 1.85 [3.09] | 0.637 [1.76] | 0.038^1^ |
| VS50 (cc), mean; [SD] | 4.0 [4.7] | 1.84 [2.84] | 0.024^1^ |
| VS60 (cc), mean; [SD] | 9.66 [9.07] | 6.71 [4.58] | 0.023^1^ |
| VS70 (cc), mean; [SD] | 15.0 [14.9] | 11.0 [4.75] | 0.006^1^ |

^1^ based on Mann-Whitney test

^2^ based on Fisher’s exact test
