# Supplementary material for: Dosimetric predictors of hypothyroidism in oropharyngeal cancer patients treated with intensity-modulated radiation therapy
Source: Radiat Oncol. 2014 Dec 5;9:269. doi: 10.1186/s13014-014-0269-4 (PMC4265326; doi:10.1186/s13014-014-0269-4)
Supplement: Additional file 2: Table S2. — V40 Gy Multivariate Model for all Patients. [file 13014_2014_269_MOESM2_ESM.doc]

Additional file 2: Table S2. V40Gy Multivariate Model for all Patients

| **Variable** | **Odds Ratio** | **Lower**  **95% CI** | **Upper**  **95% CI** | **p-Value** |
| --- | --- | --- | --- | --- |
| V40Gy | 1.02 | 1.00 | 1.05 | 0.030 |
| Ideal Body Weight | 0.95 | 0.89 | 1.02 | 0.15 |
| Thyroid Volume | 0.93 | 0.86 | 1.02 | 0.12 |
| Age at Treatment | 0.99 | 0.95 | 1.04 | 0.68 |
| Sex (M) | 0.87 | 0.12 | 6.1 | 0.89 |
